# Patterns of HIV-1 Drug Resistance Observed Through Geospatial Analysis of Routine Diagnostic Testing in KwaZulu-Natal, South Africa

**DOI:** 10.3390/v16101634

**Published:** 2024-10-19

**Authors:** Lilishia Gounder, Aabida Khan, Justen Manasa, Richard Lessells, Andrew Tomita, Melendhran Pillay, Sontaga C. Manyana, Subitha Govender, Kerri-Lee Francois, Pravi Moodley, Nokukhanya Msomi, Kerusha Govender, Raveen Parboosing, Sikhulile Moyo, Kogieleum Naidoo, Benjamin Chimukangara

**Affiliations:** 1 Department of Virology, School of Laboratory Medicine and Medical Sciences, University of KwaZulu-Natal, Durban 4001, South Africa; khana2@ukzn.ac.za (A.K.); melendhra.pillay@nhls.ac.za (M.P.); francoisk@ukzn.ac.za (K.-L.F.); moodleyp36@ukzn.ac.za (P.M.); mdlalose@ukzn.ac.za (N.M.); govenderk7@ukzn.ac.za (K.G.); raveen.parboosing@wits.ac.za (R.P.); benjiechim@gmail.com (B.C.); 2 Department of Virology, National Health Laboratory Service, Inkosi Albert Luthuli Central Hospital, Durban 4001, South Africa; subitha.govender.123@gmail.com; 3 Department of Oncology, Faculty of Medicine and Health Sciences, University of Zimbabwe, Mount Pleasant, Harare P.O. Box MP 167, Zimbabwe; jmanasa@gmail.com; 4 KwaZulu-Natal Research Innovation and Sequencing Platform (KRISP), College of Health Sciences, University of KwaZulu-Natal, Durban 4001, South Africa; lessellsr@ukzn.ac.za (R.L.); tomita@ukzn.ac.za (A.T.); 5 Centre for the AIDS Programme of Research in South Africa (CAPRISA), Durban 4001, South Africa; kogie.naidoo@caprisa.org; 6 Centre for Rural Health, School of Nursing and Public Health, University of KwaZulu-Natal, Durban 4001, South Africa; 7 Biomedical Research and Innovation Platform (BRIP), South African Medical Research Council (SAMRC), Pretoria 0001, South Africa; sontagacris@gmail.com; 8 School of Pathology, University of Witwatersrand & National Health Laboratory Service, Johannesburg 2000, South Africa; 9 Botswana Harvard Health Partnership, Gaborone P.O. Box B0320, Botswana; smoyo@bhp.org.bw; 10 Department of Immunology & Infectious Diseases, Harvard T.H. Chan School of Public Health, Boston, MA 02115, USA; 11 Department of Pathology, Division of Medical Virology, Stellenbosch University, Cape Town 7500, South Africa; 12 CAPRISA HIV-TB Pathogenesis and Treatment Research Unit, South African Medical Research Council (SAMRC), Durban 4001, South Africa; 13 Critical Care Medicine Department, NIH Clinical Center, Bethesda, MD 20892, USA

**Keywords:** HIV-1, drug resistance, KwaZulu-Natal, South Africa, geospatial analysis, dolutegravir, genotypic susceptibility scores

## Abstract

HIV-1 drug resistance (HIVDR) impedes treatment and control of HIV-1, especially in high-prevalence settings such as KwaZulu-Natal (KZN) province, South Africa. This study merged routine HIV-1 genotypic resistance test (GRT) data with Geographic Information Systems coordinates to assess patterns and geographic distribution of HIVDR in KZN, among ART-experienced adults with virological failure. We curated 3133 GRT records generated between 1 January 2018 and 30 June 2022, which includes the early phase of dolutegravir (DTG) rollout, of which 2735 (87.30%) had HIVDR. Of the 2735, major protease, nucleoside, and non-nucleoside reverse transcriptase inhibitor mutations were detected in 41.24%, 84.97% and 88.08% of GRTs, respectively. Additional genotyping of HIV-1 *integrase* for 41/3133 (1.31%) GRTs showed that 17/41 (41.46%) had integrase strand transfer inhibitor resistance. Notably, of 26 patients on DTG with *integrase* genotyping, 9 (34.62%) had DTG-associated resistance mutations. Dual- or triple-class resistance was observed in four of every five GRTs. The odds of HIVDR increased significantly with age, with ≥60 years having 5 times higher odds of HIVDR compared to 18–29 years (*p* = 0.001). We identified geospatial differences in the burden of HIVDR, providing proof of concept that this could be used for data-driven public health decision making. Ongoing real-time HIVDR surveillance is essential for evaluating the outcomes of the updated South African HIV treatment programme.

## 1. Introduction

The HIV-1 epidemic in sub-Saharan Africa is associated with a heterogeneous geographical distribution, and specific “hotspots” have gained prominence for higher HIV-1 prevalence [1]. In South Africa, where HIV-1 subtype C is predominant, the province of KwaZulu-Natal (KZN) has the highest prevalence of HIV-1 and is often regarded as the epicentre of the epidemic [2,3]. In efforts to curb the epidemic globally, HIV-1 treatment programmes have adopted the UNAIDS 95-95-95 strategy, the third 95 being 95% of people living with HIV (PLWH) on antiretroviral therapy (ART) achieving viral suppression [4]. A major obstacle to the control of HIV-1 is the development and transmission of HIV-1 drug resistance (HIVDR), with an evident increase in levels of non-nucleoside reverse transcriptase inhibitor (NNRTI) pre-treatment drug resistance (PDR) locally and globally [5,6,7,8,9,10]. In response, South Africa adopted new HIV treatment guidelines, which included a fixed-dose combination containing dolutegravir (DTG), a potent integrase strand transfer inhibitor (INSTI) with a high genetic barrier to resistance, as the preferred first-line regimen for PLWH initiating ART [11,12].

Coupled with the transition to DTG is the effective monitoring of HIVDR and treatment outcomes [13], and the ADVANCE study highlighted this by demonstrating the impact of PDR on the efficacy of first-line regimens containing DTG [14,15]. HIV viral load (VL) testing remains standard of care for monitoring patients on ART. When two or more VLs are ≥1000 copies/millilitre (mL) taken ≥2 years after starting a drug regimen containing DTG or a protease inhibitor (PI), with adherence >80%, the patient is considered to have virological failure [11]. In South Africa, as per clinical guidelines, HIVDR testing is reserved primarily for patients failing second- or third-line ART prior to switching HIV-1 treatment regimens. However, HIVDR testing for patients failing a first-line DTG-based regimen may be authorized by an expert (i.e., clinical virologist, third-line committee member, helpline consultant, or nominated provincial expert) on a case-by-case basis [11,16].

Within KZN, high HIV incidence rates persist in specific areas, reflecting the need for individualized interventions that are specific to the local epidemiology [5]. Previous work from our group using spatiotemporal mapping of HIV VLs across KZN province from 2018 to 2022, showed persistently higher VLs in northern and coastal regions of the province, despite an overall viral suppression rate of 86% [17]. Moreover, with the continuing evolution of drug resistance even after years on successful ART [18], it is imperative to have a high index of suspicion for HIVDR in older PLWH [19,20,21]. The problem of drug resistance has been described in our setting for other infectious diseases such as tuberculosis (TB), with several studies highlighting the spatial distribution of rifampicin-resistant TB within various regions of South Africa [22,23,24,25]. However, there is a paucity of scientific evidence for monitoring HIVDR through geospatial analysis in hyper-endemic areas, such as South Africa. To address the gaps in monitoring and analysis of HIVDR in KZN, we combined routine HIVDR genotypic data with Geographic Information Systems (GIS) coordinates to assess the geographic distribution and provide “real-world” estimates of patterns of HIVDR in KZN, South Africa.

## 2. Materials and Methods

### 2.1. Study Population and Design

This study was a retrospective analysis of routinely processed HIV genotypic resistance test (GRT) data acquired from the Central Data Warehouse (CDW) within the South African National Health Laboratory Service (NHLS). The NHLS is the biggest provider of diagnostic laboratory services in South Africa, and it delivers efficient and economical services to all healthcare facilities within the public sector, catering for nearly 80% of the overall population [26]. Patient demographic data and linked laboratory test data are stored at the NHLS CDW and are essential for monitoring Department of Health programmes at the national and provincial level [27].

**Figure 1:**
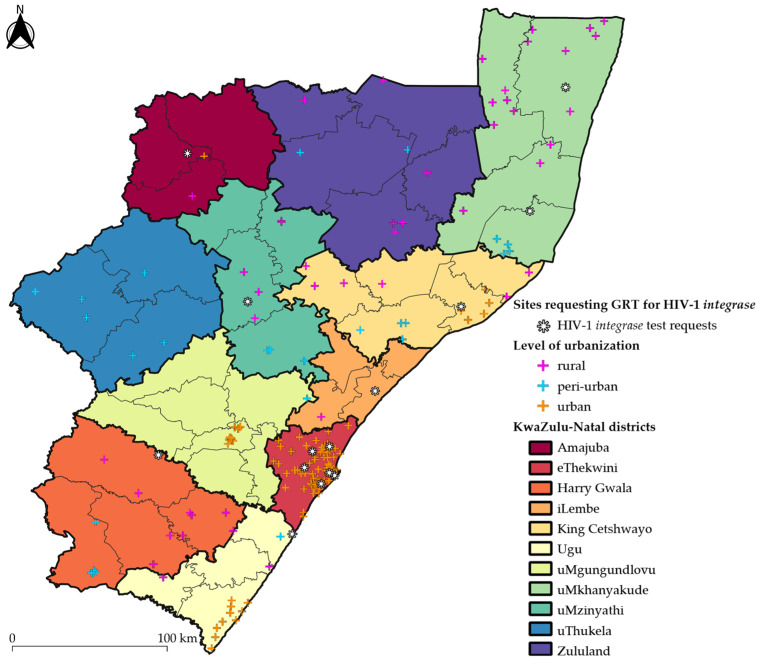
Public-sector healthcare facilities with HIV-1 genotypic resistance tests included by district in KwaZulu-Natal province, South Africa. Individual cross symbols pinpoint the Geographic Information Systems (GIS) coordinates of each healthcare facility that had requested a genotypic resistance test (GRT), the data of which had been included in the study. The asterisk denotes healthcare facilities that requested HIV-1 *integrase* testing. The cross symbols are coloured in pink, blue or orange based on whether the healthcare facility’s GIS coordinates are within a rural, peri-urban or urban subdistrict, respectively. The thin and thick black outlines represent the borders of the subdistricts and districts, respectively. Each district is illustrated in a different colour. The basemap of KwaZulu-Natal province was republished under a CC BY license with permission obtained from Carto Builder user Lilishia Gounder, original copyright 2024. Available at: https://pinea.app.carto.com/map/4d4c56c1-f82d-4409-b190-ea9ced309005 (accessed on 18 October 2024).

We obtained de-identified GRT data for PLWH ≥18 years of age who had attended a healthcare facility within the public sector in KZN province, South Africa (Figure 1). We did not include children (<18 years of age) because paediatric ART regimens differ from adult ART regimens [28] and would require a separate statistical and geospatial analysis. The study timeframe of 1 January 2018 to 30 June 2022 represented a period during the rollout and transition to DTG in first-line ART regimens in South Africa and paralleled our previous study on geospatial mapping of HIV VLs in KZN province [17]. DTG was rolled out as first-line ART in South Africa in December 2019; however, ART initiations onto TLD (Tenofovir disoproxil fumarate-Lamivudine-Dolutegravir) were slow, due to the lockdowns experienced during the COVID-19 pandemic [29]. All GRT requests and specimens were received at the Department of Virology, which is located within the NHLS laboratory division of the Inkosi Albert Luthuli Central Hospital in Durban, KZN, South Africa. The Department of Virology is the sentinel testing site for HIV GRTs in KZN province. GRT requests were clinically indicated in patients who were on PI- and/or INSTI-based ART according to the standard of care in South Africa, with VLs ≥1000 HIV-1 RNA copies/mL of plasma.

### 2.2. Routine Laboratory Procedures for HIV Genotyping

We extracted viral RNA from 1mL of patient plasma using a NucliSENS EasyMag (bioMérieux, Marcy l’Etoile, France) extraction system, according to the manufacturer’s instructions. We performed reverse transcription and nested polymerase chain reaction (PCR) amplification of the *protease* (PR) and *reverse transcriptase* (RT) genes using the Applied Biosystems HIV-1 Genotyping Kit (ThermoFisher Scientific, Waltham, MA, USA). We reversed transcribed and amplified the *integrase* (IN) gene using Superscript III one-step RT-PCR system with HiFi Platinum Taq (ThermoFisher Scientific, Waltham, MA, USA) and conducted nested PCR using the Expand High Fidelity Plus PCR System (Merck Lifesciences, Rockville, MD, USA). We verified the successful amplification of an approximately 1050 base pair (bp) fragment for PR and RT genes, and a 1200 bp fragment for the IN gene and viewed the application products on 1% agarose gel.

We performed Sanger sequencing reaction preparations using Big-Dye XTerminator v3.1 kit (Applied Biosystems, Foster City, CA, USA) for PR, RT and IN genes, according to the manufacturer’s instructions. We sequenced samples on an ABI 3730 Genetic Analyzer (Applied Biosystems, Foster City, CA, USA) using PR and RT sequencing primers from Applied Biosystems HIV-1 Genotyping Kit (ThermoFisher Scientific, Waltham, MA, USA), and in-house IN sequencing primers described previously [30,31]. We assessed sequence quality and generated consensus sequence alignments using Geneious Prime software 2021.1.1 (Biomatters Ltd., Auckland, New Zealand). We detected HIVDR mutations and determined antiretroviral (ARV) drug susceptibilities using the Stanford University HIV Drug Resistance Database v9.0. [32].

We determined PI drug susceptibilities for ritonavir-boosted atazanavir (ATV/r), darunavir (DRV/r), and lopinavir (LPV/r). Drug susceptibilities for nucleoside/nucleotide reverse transcriptase inhibitors (NRTIs), NNRTIs and INSTIs included lamivudine (3TC), abacavir (ABC), zidovudine (AZT), emtricitabine (FTC), tenofovir disoproxil fumarate (TDF), efavirenz (EFV), etravirine (ETR), nevirapine (NVP), raltegravir (RAL), and DTG. We defined HIVDR as any GRTs with the following ARV drug susceptibilities: potential low-level resistance (PLLR), low-level resistance (LLR), intermediate resistance (IR), or high-level resistance (HLR) to any PI, NRTI, NNRTI or INSTI available as per standard of care in South Africa.

### 2.3. Interactive HIV-1 Drug Resistance Database

We created an interactive database using HIVDR reports from PLWH ≥18 years of age with HIV GRT data generated during the period from 1 January 2018 to 30 June 2022, at healthcare facilities within the public sector in KZN province. We categorized GRT records into the following two groups: (i) HIVDR, resulting in reduced susceptibility to at least one ARV drug, and (ii) no HIVDR, resulting in full susceptibility to ARV drugs. We included age, sex, specimen collection date, district of specimen origin, healthcare facility name, healthcare facility type (i.e., outpatient or inpatient department), healthcare facility level of urbanization, HIVDR mutations detected, ARV drug susceptibility, current ARV drug regimen, and most recent HIV VL, as meta-data for each GRT database record. We categorized age into 3 groups: (i) young adults (18–29 years), (ii) adults (30–59 years), and (iii) elderly (≥60 years). We excluded GRT records where the patient’s age was <18 years or the patient’s age was not included. Based on the healthcare facility location, we assigned global positioning system (GPS) coordinates to each GRT record.

We further assigned genotypic susceptibility scores (GSSs) to GRT records with complete regimen data. For each drug prescribed, a GSS value of 1 was assigned if no resistance or PLLR was determined, while 0.5 was assigned if resistance was LLR or IR, and 0 was assigned if HLR was determined. The sum of all the individual drug scores provided the total GSS for that regimen. Patients who had complete drug regimen data were grouped based on the number of active drugs prescribed: <2; 2; and >2. A value of >2 indicated an active regimen, whereas values of <2 indicated drug resistance with reduced potency of the ARV regimen [33].

### 2.4. Statistical Analysis

We used chi-square tests and logistic regression analysis to assess associations between HIVDR and sex, year of specimen collection, ARV drug regimen, healthcare facility type, level of urbanization, age group, and HIV VLs. We used descriptive statistics to analyze patterns of drug class resistance, genotypic susceptibility scores and mutations, presented as proportions/percentages in graphs and tables. We performed all statistical analyses using Stata 18.0 software SE (StataCorp. 2023, College Station, TX, USA).

### 2.5. Geospatial Mapping of HIV Genotypic Resistance Test Records

Using healthcare facility-linked GPS coordinates from the curated HIV GRT database, we generated geospatial maps in QGIS 3.30 software [34], with each GRT record assigned to the healthcare facility of specimen collection. We created inverse distance weighted (IDW) interpolation maps to visualize the ARV drug susceptibility levels per healthcare facility. With the IDW interpolation method, unsampled areas closest to the healthcare facility show values comparable to the value measured at the healthcare facility location.

## 3. Results

**Figure 2:**
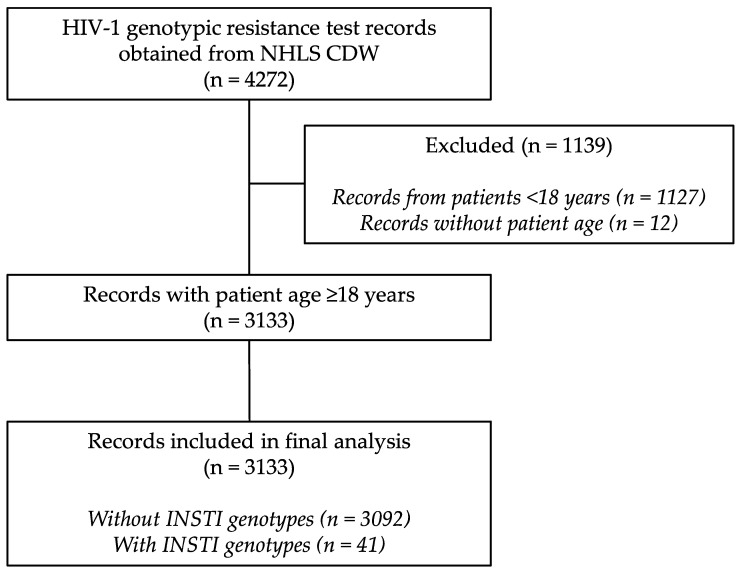
Flow diagram of HIV-1 genotypic resistance test records obtained and included in the final analysis. CDW, central data warehouse; INSTI, integrase strand transfer inhibitor; NHLS, National Health Laboratory Service.

We curated 3133 GRT records from 179 public-sector healthcare facilities across the 11 districts of KZN province (Supplementary Figure S1 and Table S1), of which 2735 (87.30%) had HIVDR. Additionally, HIV-1 *integrase* genotyping was requested in 41/3133 (1.31%) GRT records. Only 2 out of the 41 GRTs with *integrase* genotyping were fully susceptible to all ARV drugs. About 9 in every 10 GRTs were from patients on PI-based ART (2830/3133) with either LPV/r or ATV/r. Figure 2 shows a summary of GRT records that were obtained and analysed.

More than two thirds (2202/3133; 70.28%) of GRTs were from healthcare facilities within urban subdistricts, with nearly similar proportions from peri-urban (480/3133; 15.32%) and rural (451/3133; 14.40%) subdistricts (Table 1).

Detection of HIVDR was 90.24%, 86.42% and 88.54% in facilities within rural, urban and peri-urban subdistricts, respectively. The GRTs that included HIV-1 *integrase* genotyping were predominantly (33/41; 80.49%) from healthcare facilities within urban subdistricts. There was a higher proportion of GRTs from females (1982/3133; 63.26%) as compared to males (1126/3133; 35.94%). Relative to those in the age group 18–29 years, the odds of HIVDR were more than 2 times higher among those aged 30–59 years (odds ratio (OR) = 2.27, 95% confidence interval (CI): 1.82–2.83, *p* < 0.001), and nearly 5 times higher among those aged ≥60 years (OR = 4.94, 95% CI: 1.97–12.35, *p* = 0.001). For every log_10_ VL increase, there was a 31.28% reduction in the odds of HIVDR detection (OR = 0.687, 95% CI: 0.60–0.79, *p* < 0.001). The odds of HIVDR detection did not significantly change by year of collection, ARV drug regimen, healthcare facility type or facility level of urbanization (*p* > 0.05) (Table 1).

### 3.1. Patterns of HIV-1 Drug Resistance

#### 3.1.1. Drug Class Resistance

**Figure 3:**
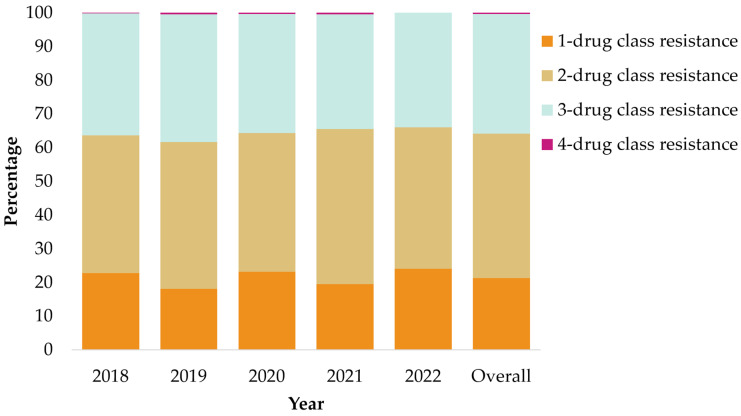
Patterns of antiretroviral drug class resistance observed in 2735 genotypes with HIVDR obtained from KwaZulu-Natal province, South Africa. Please note that 41 genotypes included HIV-1 *integrase* testing, of which only 9 met the definition for 4-drug class resistance.

Of the 2735 (87.30%) with HIVDR detected, 583 (21.32%) had single-class resistance, 1170 (42.78%) had dual-class resistance, 973 (35.58%) had triple-class resistance, and 9 (0.33%) had HIVDR to four-drug classes (Figure 3), of which 3/9 were on DTG-based ART. The predominant pattern of resistance identified in approximately two-thirds of GRTs, was NRTI and NNRTI drug class resistance, either with PI resistance (35.36%) or without PI resistance (37.80%). The frequency of HIVDR mutations by drug class was as follows: 1128 (41.24%) with major PI-mutations, 2324 (84.97%) with NRTI mutations, and 2409 (88.08%) with NNRTI mutations. Only 41 GRTs had HIV-1 *integrase* resistance testing conducted, of which 39 had HIVDR mutations, with 17 (43.59%) having INSTI-specific mutations. Overall, there were no changes in levels of resistance observed over time. The patterns and proportions of drug class resistance observed per year are shown in Figure 3 and Supplementary Table S2.

#### 3.1.2. Mutations Detected in Genotypic Resistance Test Records with HIV-1 Drug Resistance

**Figure 4:**
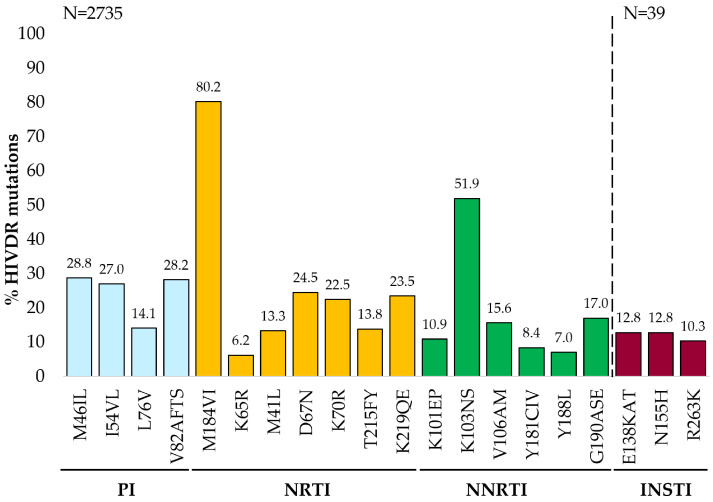
Specific mutations detected in 2735 genotypes with HIVDR obtained from KwaZulu-Natal province, South Africa. Mutations shown on the horizontal axis include “major” mutations observed in >6% of the genotypes with HIV-1 drug resistance, “major” as defined by Stanford HIV Drug Resistance Database or 2022 edition IAS–USA drug resistance mutations list [32,35]. HIVDR, HIV-1 drug resistance; INSTI, integrase strand transfer inhibitor; NNRTI, non-nucleoside reverse transcriptase inhibitor; NRTI, nucleoside reverse transcriptase inhibitor; PI, protease inhibitor.

The most frequently observed mutations by drug class were NRTI mutation M184VI (80.2%), NNRTI mutation K103NS (51.9%), and PI mutation M46IL (28.8%). Of the 39 *integrase* genotypes with HIVDR mutations, E138KAT and N155H (12.8%) were the most common INSTI mutations detected (Figure 4). Approximately half (1161/2324; 49.96%) of all GRTs with NRTI mutations had thymidine analogue mutations (TAMs). Overall, the most common TAMs observed were type-2 TAMs D67N (670/2735), K219QE (642/2735), and K70R (614/2735), and the type-1 TAM M41L (364/2735). The K65R mutation was detected in 6.22% (170/2735) of GRTs and 37.06% (63/170) of those with K65R had received TDF in their current regimen. Of 699 patients on TDF-based regimens, 295 had TAMs. Of those, we had data on prior ARV exposure for 270/295, and 210 of the 270 (77.77%) had documented prior drug exposure to thymidine analogues, i.e., AZT or stavudine.

Despite only 36 (1.15%) GRTs from patients on current NNRTI-based ART, approximately 8 in every 9 (2409/2735) GRTs had NNRTI-associated HIVDR mutations detected. In addition to K103NS, the G190ASE, V106AM, and K101EP mutations were each detected in more than 10% of GRT requests. The proportion of GRTs with intermediate to high-level ETR resistance was 20.99% (574/2735), of which 67.07% (385/574) had ETR-associated mutations, i.e., L100I, K101P, Y181CIV, G190E and M230L. Overall, the mutation E138AGKQ conferred resistance to ETR in 13.67% (374/2735) of HIVDR GRTs.

Of the 1128 GRTs with major PI mutations, 894 (79.26%) had intermediate to high-level resistance to LPV/r or ATV/r, with two-thirds (588/894) also conferring resistance to DRV/r. However, the majority (2182/2735; 79.78%) of HIVDR GRTs showed susceptible to potential low-level resistance to DRV/r. Twenty-six of thirty-nine INSTI GRTs were from patients on DTG, of which 9 (34.62%) had mutations that alone confer DTG resistance, namely, H51Y, G118R, E138KAT, G140A, S147G, Q148R, N155H, and R263K (Supplementary Table S3). Details of mutations detected in all GRTs with HIVDR were categorized by drug class [32,35] and are listed in Supplementary Table S4.

#### 3.1.3. Genotypic Susceptibility Scores

For the purposes of genotypic susceptibility score (GSS) analysis, we included only GRTs that had complete regimen data available (i.e., 2934/3133), regardless of whether they had HIVDR or not. Of the 2934 GRTs, 46 were from patients receiving DTG in their current ART regimen (Table 2). All current regimens included XTC, which is either 3TC or FTC. Patients on EFV-based ART with TDF had the lowest predicted GSSs, with approximately 6 in every 7 having a GSS of <2. Other regimens with lower predicted GSSs included being on a RAL regimen with TDF, a DRV/r regimen with TDF, an ABC regimen with a boosted PI, and an ABC regimen with DTG. Generally, 3-drug regimens with DTG had poor GSSs, with most having a GSS ≤ 2 (Table 2). The highest GSSs (>2) were predicted in regimens that included at least four drugs, with TDF and AZT, plus DTG and/or DRV/r. Table 2 shows a summary of the regimens that are grouped based on their NRTI drugs, which included TDF-based, AZT-based, TDF plus AZT-based, and ABC-based ART.

### 3.2. Geospatial Analysis of HIV-1 Drug Resistance

**Figure 5:**
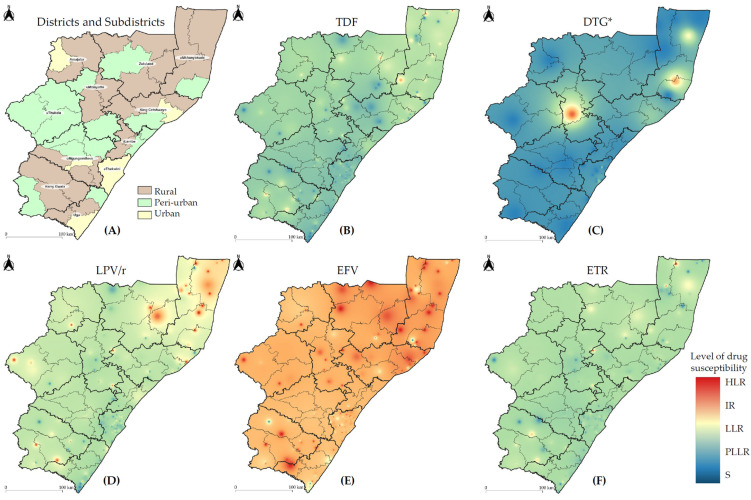
Antiretroviral drug susceptibility levels across KwaZulu-Natal province, South Africa. Districts and subdistricts are categorized as follows: (**A**) level of urbanization. Inverse distance weighted interpolation maps cumulatively reflect the drug susceptibilities for the following: (**B**) TDF, tenofovir; (**C**) DTG, dolutegravir; (**D**) LPV/r, lopinavir with boosted ritonavir; (**E**) EFV, efavirenz; (**F**) ETR, etravirine. Spectral colour change from blue to red reflects the drug susceptibility level as follows: S, susceptible; PLLR, potential low-level resistance; LLR, low-level resistance; IR, intermediate resistance; HLR, high-level resistance. The thin and thick black outlines represent the borders of the 44 subdistricts and 11 districts of KwaZulu-Natal (KZN) province, respectively. The basemap of KZN province was republished under a CC BY license with permission obtained from Carto Builder user Lilishia Gounder, original copyright 2024. Available at: https://pinea.app.carto.com/map/4d4c56c1-f82d-4409-b190-ea9ced309005 (accessed on 18 October 2024). * Please note that HIV-1 *integrase* testing was performed for 41 genotypes; the remaining 3092 genotypes that did not have *integrase* test requests were assumed to be susceptible to DTG for the purposes of creating the DTG interpolation map.

ARV drug susceptibility levels were mapped by facility location across KZN province to assess the impact on select drugs of interest in South Africa, as shown in Figure 5 and Supplementary Figure S2. Generally, most districts had susceptible to low-level resistance to TDF and ETR, with slightly higher levels of resistance in northern KZN. Low to intermediate levels of LPV/r resistance were also noted in northern KZN, with susceptible to potential low-level resistance to DRV/r, a drug of choice in people failing DTG-based ART (Supplementary Figure S2). All districts showed intermediate to high levels of resistance to EFV, NVP, and XTC, despite most GRTs (93.16%) being obtained from patients on non-NNRTI-based regimens. Again, northern KZN showed the highest burden of EFV, NVP, and XTC resistance across the province (Figure 5, Supplementary Figure S2). The geospatial analysis of DTG susceptibility levels was limited, due to the small number (41/3133) of HIV-1 *integrase* genotypes requested. Low- to high-level DTG resistance was noted in two rural districts, based only on two DTG-resistant genotypes from either district.

## 4. Discussion

In this retrospective analysis of more than 3000 routine HIV genotypic data from KZN province, South Africa, we observed high proportions of HIVDR among ART-experienced adults with virological failure, consistent with previous studies [36,37]. This was mainly driven by *reverse transcriptase* mutations, with approximately 1 in every 3 GRTs having dual-class NRTI and NNRTI resistance. The majority of GRTs (90%) were from patients currently receiving a PI-based regimen, but only 36% (1128/3133) had PI resistance, an attribute of PI’s high genetic barrier to resistance [38,39], and suggesting that virological failure on LPV/r-based regimens is driven by poor adherence related to drug tolerability. Of those on DTG-based ART with an INSTI genotype, ~35% had DTG-associated resistance, raising concerns about the durability of DTG in highly treatment-experienced patients. In addition, geospatial analysis showed higher levels of resistance to EFV and other drugs, particularly in northern rural KZN, a known HIV hyper-endemic region [3,5], highlighting the need for intensified HIV-1 treatment monitoring through regular VL testing, adherence support interventions, ongoing drug resistance surveillance, and strengthening of health systems. This study serves as a proof of concept that geospatial analysis could potentially be used for data-driven public health decision making.

South Africa rolled out DTG in the National HIV treatment programme in December 2019 [11]. Amid this transition, our study found persistently increased levels of NNRTI and NRTI resistance in patients with virological failure, similar to other South African studies [5,6,7,36,40]. However, in this study, of 26 patients on DTG with an INSTI genotype, ~35% (9/26) had DTG-associated resistance mutations, which is among the highest reported to date. The DTG-RESIST study among viraemic adults on DTG-based ART observed ~5% DTG resistance [41,42]. More cross-sectional studies from low-to-middle-income countries are showing levels of DTG resistance ranging from ~4 to 20% among people with virological failure on DTG-based ART, exceeding levels reported in clinical trials, even among highly treated individuals, as observed by the World Health Organization [42,43]. In this study, the most common mutation observed in the nine patients with DTG-associated resistance was E138KAT (4/9), which alone confers potential low-level resistance to DTG and low-level resistance to cabotegravir (CAB) [32]. Other mutations included G118R (3/9) and R263K (3/9), the most common DTG resistance mutations in INSTI-naïve individuals with virological failure [44], conferring high- and intermediate levels of resistance to DTG, respectively [32]. We also observed the occurrence of multiple INSTI resistance mutations in one individual (T66A, E138K, Y143R, with accessory mutations Q95K, T97A and S147), which together cause low- to high-level resistance to all FDA-approved INSTI drugs [32]. Although our limited geospatial analysis for DTG indicates high viral susceptibility levels across the KZN province, the accumulation of mutations in those with INSTI resistance signify an impending problem and suggests the need for heightened surveillance of DTG-specific mutations to avoid jeopardizing future DTG-based ART, long-acting CAB-based treatment, and prevention strategies.

Approximately 89% of all HIVDR GRTs had NNRTI-associated mutations detected, despite only 1% on current NNRTI-based regimens, showing the persistence of NNRTI mutations. One in every five GRTs had intermediate to high-level resistance to ETR, a second-generation NNRTI, of which 67% had mutations that confer resistance to both first- and second- generation NNRTIs, i.e., L100I, K101P, Y181CIV, G190E and M230L. The NNRTI mutation E138AGKQ is considered a polymorphism in HIV-1 subtype C, but it conferred resistance to ETR in 13.67% (374/2735) of HIVDR GRTs, warranting further evaluation of the impact of *reverse transcriptase* mutations at position 138 on ETR treatment [45,46]. Despite EFV being largely compromised, geospatial analysis in this study shows relatively lower levels of resistance to ETR across KZN, warranting its continued use in future regimens.

The temporal trends of HIVDR were similar, with dual- or triple-class resistance observed in four out of every five patients. The increased risk of virological failure driven by the presence of dual-class resistance has been described previously [47]. Half of all GRTs with NRTI mutations detected had at least one TAM. The proportion of TAMs identified in GRTs from patients on TDF-based regimens with documented exposure to thymidine analogues was 77.77% (210/270), suggestive of accumulation of TAMs during prior ART exposure. By inference, proportions of atypical TAMs were ~22.22%; however, this may not be an accurate reflection since we do not know whether the prior drug exposure data for these patients were complete. Other studies from sub-Saharan Africa reported prevalences ranging from 16 to 41% for atypical TAMs [37,48], and considering their potential to reduce susceptibility to AZT and TDF, there is a need to better understand the occurrence of atypical TAMs in TDF-treated patients. The K65R mutation, which increases viral susceptibility to AZT and reduces viral susceptibility to TDF, was present in over 6% of GRTs, as shown in the geospatial analysis where most regions in KZN show susceptible to low levels of TDF resistance. Cumulatively, 699 GRTs were from patients on a regimen containing TDF, and of those 43.78% (306/699) had a GSS of <2. Other studies have reported higher proportions of K65R mutations in the South African setting [37,49].

The value of geospatial data visualization techniques in improving understanding of the spatial distribution of HIV and resistance mutation prevalence has been previously described [5]. However, this study used geospatial data visualization at a much more granular level and based on community surveillance. In our study, geospatial analysis of drug susceptibility levels mapped across KZN demonstrated that northern KZN had the highest burden of EFV, NVP, and XTC resistance, albeit high levels of resistance evident in most parts of the province. Low to intermediate levels of LPV/r and DTG resistance were also noted in northern KZN, highlighting a need for targeted programmatic interventions in this area of the province. Although two-thirds of GRTs were from patients who had attended healthcare facilities in urban subdistricts, more HIVDR was detected in rural areas. Rural areas have fewer resources available and less consistent monitoring of HIV as compared to urban settings [50], and other studies in rural settings have shown increasing proportions of HIVDR [51]. Taken together, these data suggest the need for vigilant HIVDR monitoring in patients experiencing virological failure on first-line TDF + XTC + DTG, with timely HIVDR genotyping and switching of ART, among second-line failures.

Although more GRTs were obtained from females, the levels of HIVDR were similar among males and females. The absence of HIVDR and the presence of high VLs in those aged 18–59 years implies non-adherence, as evident in other studies with similar age groups [50,52], and interventions must be prioritized in this population to curb the progression to HIVDR. Other studies have described the relationship between HIVDR and older age, even highlighting the role of elderly PLWH in the transmission of PDR [19,20,21]. While it is possible that older patients were more treatment-experienced and more likely to be exposed to drug interactions with ART from treatments prescribed for other co-morbidities, these data were not available for inclusion in this analysis.

Our findings should be interpreted with consideration of the following limitations. We did not account for GRT testing practices per facility, nor contextualize our geospatial analysis within the local population of PLWH, nor ART coverage per district. Future research should aim to integrate HIV VL and GRT data, ideally within the context of ART coverage, to provide more detailed insights that inform data-driven public health decision making. The HIVDR database was based on de-identified GRTs, so we could not determine whether a patient had more than one GRT performed, and we could not link serial GRTs, if any. There were missing data for certain database records, because some patient data were not recorded on test request forms completed at sample collection. Previous ARV drug regimen data were not consistently documented, so, where available, we described prior thymidine analogue exposure for patients currently on TDF-based regimens with TAMs. Moreover, duration of ART regimen was not electronically captured; therefore, we could not contextualize the persistence of HIVDR mutations within a specific timeframe. Bearing in mind that <2% of patients were on INSTI-based regimens, appropriate population-level analyses of the impact of specific mutations were limited. If HIV-1 *integrase* testing was not requested and therefore not performed, we presumed the GRT to be susceptible to INSTI drugs. We made this presumption for the purposes of creating the DTG interpolation map, determining the number of drug-class resistance patterns and calculating the GSS scores. It has been shown that DTG resistance is rare in the general population of PLWH, especially among ART-naïve individuals [53]. Our geospatial analysis was based on facility location, not households, based on the presumption that patients sought healthcare services at facilities closest to their homes. The clinic where the GRT was collected might not be the site where the individual receives most HIV care, especially if there is an up-referral to advanced clinical care facilities for patients with virological failure. However, a previous study from South Africa demonstrated that about 67% of individuals live less than 2 kilometres away from their nearest primary healthcare facility [54].

Considering that this study was performed during the transition to DTG-based first-line regimens in South Africa, there is a need to assess the current prevalence of HIVDR mutations, particularly those associated with DTG resistance, to identify areas in need of directed interventions. In conclusion, our study demonstrated that the trends of HIVDR have remained static among treatment-experienced adults failing ART in KZN. Therefore, ongoing real-time HIVDR surveillance with geospatial mapping is essential for evaluating the outcomes of the updated HIV treatment programmes, while identifying areas with higher HIVDR, and providing directed interventions at a patient and public health level.

## Figures and Tables

**Table 1:** Characteristics of HIV-1 genotypic resistance test records included in this study.

Variable	All N = 3133 (100.00%)	No HIVDR N = 398 (12.70%)	HIVDR N = 2735 (87.30%)
**Sex**			
Male	1126 (35.94%)	130 (11.55%)	996 (88.45%)
Female	1982 (63.26%)	266 (13.42%)	1716 (86.58%)
Unknown	25 (0.80%)	2 (8.00%)	23 (92.00%)
**Age** in years, median (IQR)	39 (30–46)	36 (22–43)	39 (32–46)
18–29	750 (23.94%)	156 (20.80%)	594 (79.20%)
30–59	2284 (72.90%)	237 (10.38%)	2047 (89.62%)
≥60	99 (3.16%)	5 (5.05%)	94 (94.95%)
**Collection year**			
2018	597 (19.06%)	75 (12.56%)	522 (87.44%)
2019	675 (21.54%)	78 (11.56%)	597 (88.44%)
2020	763 (24.35%)	115 (15.07%)	648 (84.93%)
2021	666 (21.26%)	72 (10.81%)	594 (89.19%)
2022 ^a^	432 (13.79%)	58 (13.43%)	374 (86.57%)
**ARV drug regimen**			
LPV/r or ATV/r -based	2830 (90.33%)	362 (12.79%)	2468 (87.21%)
DRV/r-based ^b^	30 (0.96%)	1 (3.33%)	29 (96.67%)
DRV/r-based with RAL	9 (0.29%)	0 (0.00%)	9 (100%)
DRV/r-based with DTG	10 (0.32%)	1 (10.00%)	9 (90.00%)
RAL-based ^c^	5 (0.16%)	1 (20.00%)	4 (80.00%)
DTG-based ^c^	36 (1.15%)	7 (19.44%)	29 (80.56%)
NNRTI-based ^d^	36 (1.15%)	5 (13.89%)	31 (86.11%)
Unknown	177 (5.65%)	21 (11.86%)	156 (88.14%)
**HIV VL** in log_10_ copies/mL, median (IQR) ^e^	4.71 (4.10–5.28)	4.99 (4.33–5.45)	4.66 (4.07–5.25)
**Healthcare facility type**			
Outpatients	2950 (94.16%)	373 (12.64%)	2577 (87.36%)
Inpatients	183 (5.84%)	25 (13.66%)	158 (86.34%)
**Level of urbanization ^f^**			
Rural subdistricts	451 (14.40%)	44 (9.76%)	407 (90.24%)
Peri-urban subdistricts	480 (15.32%)	55 (11.46%)	425 (88.54%)
Urban subdistricts	2202 (70.28%)	299 (13.58%)	1903 (86.42%)

ARV, antiretroviral; ATV/r, ritonavir-boosted atazanavir; copies/mL, copies/millilitre; DRV/r, ritonavir-boosted darunavir; DTG, dolutegravir; EFV, efavirenz; HIVDR, human immunodeficiency virus drug resistance; INSTI, integrase strand transfer inhibitor; IQR, interquartile range; LPV/r, ritonavir-boosted lopinavir; NNRTI, non-nucleoside reverse transcriptase inhibitor; RAL, raltegravir; VL, viral load. ^a^ Data only include period from 1 January 2022 to 30 June 2022. ^b^ Etravirine included in 4 regimens containing ritonavir-boosted darunavir. ^c^ Regimen did not include a protease inhibitor. ^d^ Etravirine included in 1 NNRTI-based regimen. ^e^ Most recent HIV VL missing for 104 records. ^f^ Categories obtained from integrated development plans and annual reports for individual subdistricts in KwaZulu-Natal.

**Table 2:** Genotypic susceptibility scores for antiretroviral drug regimens used in South African public health sector.

		Genotypic Susceptibility Score for Regimen		
Current Regimen *	N = 2934	<2	2	>2
**TDF-based**	***n* = 649**			
TDF + XTC + EFV	20	17 (85%)	0	3 (15%)
TDF + XTC + boosted ATV/LPV	574	255 (44.42%)	111 (19.34%)	208 (36.24%)
TDF + XTC + boosted DRV	19	12 (63.16%)	4 (21.05%)	3 (15.79%)
TDF + XTC + RAL	4	3 (75%)	0	1 (25%)
TDF + XTC + DTG	19	8 (42.10%)	2 (10.53%)	9 (47.37%)
TDF + XTC + RAL + boosted DRV	6	3 (50%)	0	3 (50%)
TDF + XTC + DTG + boosted DRV	6	1 (16.67%)	2 (33.33%)	3 (50%)
TDF + XTC + DTG + boosted DRV + ETR	1	0	0	1 (100%)
**AZT-based**	***n* = 1927**			
AZT + XTC + boosted ATV/LPV	1911	761 (39.82%)	663 (34.69%)	487 (25.49%)
AZT + XTC + boosted DRV	4	0	1 (25%)	3 (75%)
AZT + XTC + DTG	10	4 (40%)	2 (20%)	4 (40%)
AZT + XTC + DTG + boosted DRV	1	0	0	1 (100%)
AZT + XTC + DTG + boosted DRV + ETR	1	0	0	1 (100%)
**TDF- and AZT-based**	***n* = 45**			
AZT + XTC + boosted ATV/LPV + TDF	37	4 (10.81%)	3 (8.11%)	30 (81.08%)
AZT + XTC + boosted DRV + TDF	6	0	0	6 (100%)
AZT + XTC + DTG + TDF	1	0	0	1 (100%)
AZT + XTC + DTG + boosted DRV + TDF	1	0	0	1 (100%)
**ABC-based**	***n* = 313**			
ABC + XTC + boosted ATV/LPV	307	199 (64.82%)	3 (0.98%)	105 (34.20%)
ABC + XTC + DTG	6	4 (66.67%)	0	2 (33.33%)

ABC, abacavir; ATV, atazanavir; AZT, zidovudine; DRV, darunavir; DTG, dolutegravir; EFV, efavirenz; ETR, etravirine; LPV, lopinavir; RAL, raltegravir; TDF, tenofovir disoproxil fumarate; XTC, lamivudine/emtricitabine. * Genotypic tests were conducted at the time of antiretroviral treatment failure. Integrase strand transfer inhibitors are assumed to have an individual genotypic susceptibility score (GSS) of 1 if HIV-1 *integrase* testing was not already included in the genotypic resistance test record. Boosted refers to ritonavir-boosted regimens. For ATV/LPV, the drug with the lower penalty score in Stanford University HIV Drug Resistance Database was used.

## Data Availability

The original contributions presented in the study are included in the article and Supplementary Materials, and further inquiries can be directed to the corresponding author.

## Supplementary Materials

The following supporting information can be downloaded at: https://www.mdpi.com/article/10.3390/v16101634/s1, Figure S1: Proportion of HIV-1 genotypic resistance tests per subdistrict in KwaZulu-Natal, South Africa; Figure S2: Interpolation maps of antiretroviral drug susceptibility levels across KwaZulu-Natal province, South Africa; Table S1: HIV-1 genotypic resistance tests categorized by the districts and subdistricts of KwaZulu-Natal province, South Africa; Table S2: Patterns of antiretroviral drug class resistance found in 3133 genotypic resistance tests from patients in KwaZulu-Natal province, South Africa; Table S3: Characteristics of nine patients on dolutegravir-based treatment with dolutegravir resistance in KwaZulu-Natal, South Africa; Table S4: HIV-1 drug resistance mutations detected in genotypic resistance tests from patients in KwaZulu-Natal, South Africa.
