# Supplementary material for: Patterns of HIV-1 Drug Resistance Observed Through Geospatial Analysis of Routine Diagnostic Testing in KwaZulu-Natal, South Africa
Source: Viruses. 2024 Oct 19;16(10):1634. doi: 10.3390/v16101634 (PMC11512327; doi:10.3390/v16101634)
Supplement: Supplementary file 1 [file viruses-16-01634-s001.zip › Supplementary Figure S1.pdf]

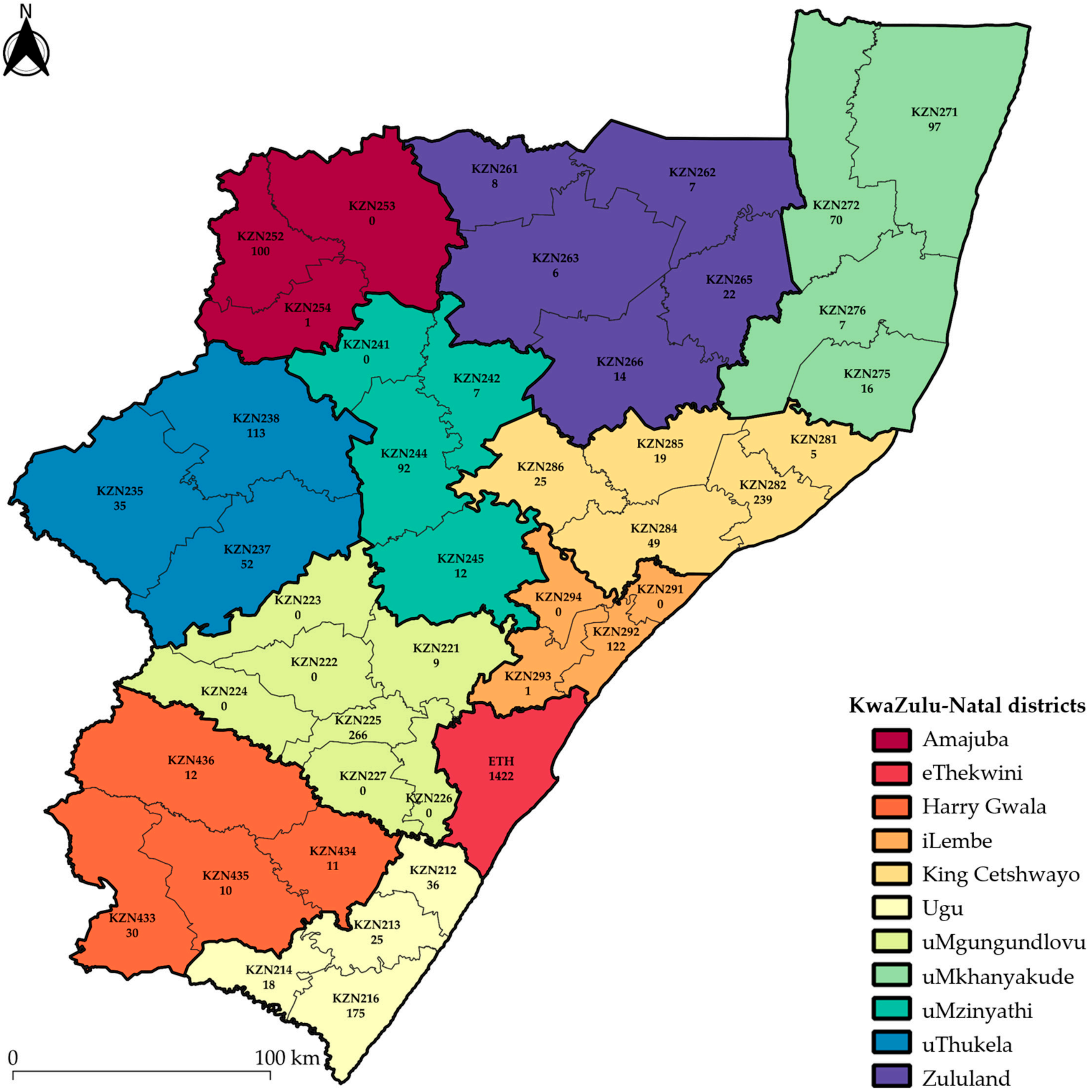

Supplementary Figure S1. Proportion of HIV-1 genotypic resistance tests per subdistrict in KwaZulu-Natal, South Africa. The sub-district name together with the total number of genotypes received from that respective subdistrict, is shown in black text on the map. The thin and thick black outlines represent the borders of the subdistricts and districts, respectively. Each district is illustrated in a different colour. The basemap of KwaZulu-Natal province was republished under a CC BY license with permission obtained from Carto Builder user Lilishia Gounder, original copyright 2024. Available at: https://pinea.app.carto.com/map/4d4c56c1-f82d-4409-b190-ea9ced309005.
