# Supplementary material for: Patterns of HIV-1 Drug Resistance Observed Through Geospatial Analysis of Routine Diagnostic Testing in KwaZulu-Natal, South Africa
Source: Viruses. 2024 Oct 19;16(10):1634. doi: 10.3390/v16101634 (PMC11512327; doi:10.3390/v16101634)
Supplement: Supplementary file 1 [file viruses-16-01634-s001.zip › Supplementary Figure S2.pdf]

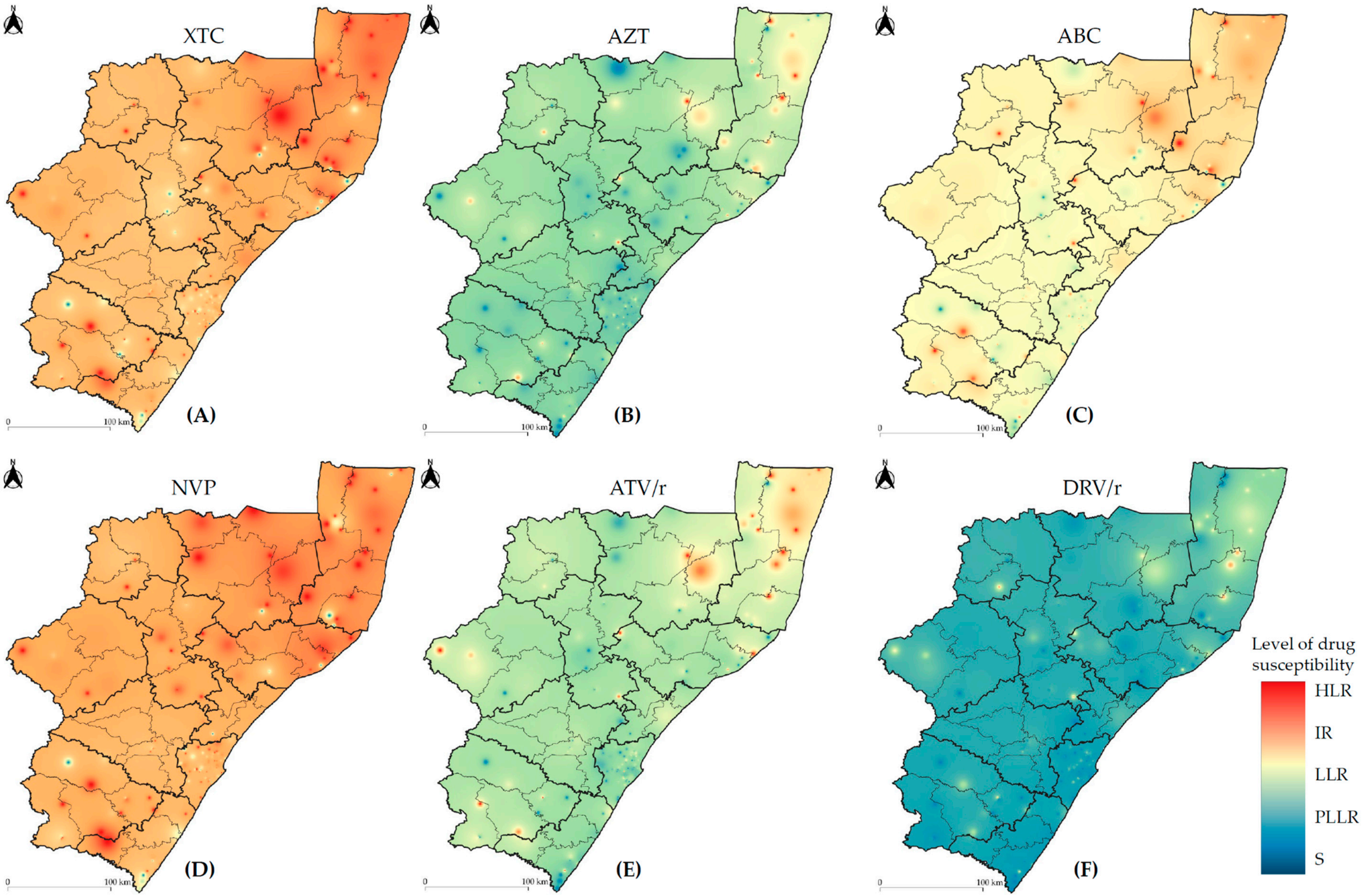

Supplementary Figure S2. Interpolation maps of antiretroviral drug susceptibility levels across KwaZulu-Natal province, South Africa. Inverse distance weighted interpolation maps cumulatively reflect the drug susceptibilities for: (**A**) XTC, lamivudine or emtricitabine; (**B**) AZT, zidovudine; (**C**) ABC, abacavir; (**D**) NVP, nevirapine; (**E**) ATV/r, atazanavir with boosted ritonavir and (**F**) DRV/r, darunavir with boosted ritonavir. Spectral colour change from blue to red reflects the drug susceptibility level as follows: S, susceptible; PLLR, potential low-level resistance; LLR, low-level resistance; IR, intermediate resistance; HLR, high-level resistance. The thin and thick black outlines represent the borders of the 44 subdistricts and 11 districts of KwaZulu-Natal (KZN) province, respectively. The basemap of KZN province was republished under a CC BY license with permission obtained from Carto Builder user Lilishia Gounder, original copyright 2024. Available at: https://pinea.app.carto.com/map/4d4c56c1-f82d-4409-b190-ea9ced309005.
