# Supplementary material for: Patterns of HIV-1 Drug Resistance Observed Through Geospatial Analysis of Routine Diagnostic Testing in KwaZulu-Natal, South Africa
Source: Viruses. 2024 Oct 19;16(10):1634. doi: 10.3390/v16101634 (PMC11512327; doi:10.3390/v16101634)
Supplement: Supplementary file 1 [file viruses-16-01634-s001.zip › Supplementary Table S1.pdf]

Supplementary Table S1. HIV-1 genotypic resistance tests categorized by the districts and subdistricts of KwaZulu-Natal province, South Africa.

District	Subdistrict	Total no. of GRTs	GRTs without HIVDR	GRTs with HIVDR
eThekwini	ETH	1422 (45.39%)	204 (14.35%)	1218 (85.65%)
Ugu	KZN212	36 (1.15%)	5 (13.89%)	31 (86.11%)
Ugu	KZN213	25 (0.80%)	5 (20.00%)	20 (80.00%)
Ugu	KZN214	18 (0.57%)	0 (0.00%)	18 (100.00%)
Ugu	KZN216	175 (5.59%)	27 (15.43%)	148 (84.57%)
uMgungundlovu	KZN221	9 (0.29%)	2 (22.22%)	7 (77.78%)
uMgungundlovu	KZN225	266 (8.49%)	32 (12.03%)	234 (87.97%)
uThukela	KZN235	35 (1.12%)	4 (11.43%)	31 (88.57%)
uThukela	KZN237	52 (1.66%)	8 (15.38%)	44 (84.62%)
uThukela	KZN238	113 (3.61%)	13 (11.50%)	100 (88.50%)
uMzinyathi	KZN242	7 (0.22%)	2 (28.57%)	5 (71.43%)
uMzinyathi	KZN244	92 (2.94%)	12 (13.04%)	80 (86.96%)
uMzinyathi	KZN245	12 (0.38%)	1 (8.33%)	11 (91.67%)
Amajuba	KZN252	100 (3.19%)	16 (16.00%)	84 (84.00%)
Amajuba	KZN254	1 (0.03%)	0 (0.00%)	1 (100.00%)
Zululand	KZN261	8 (0.26%)	0 (0.00%)	8 (100.00%)
Zululand	KZN262	7 (0.22%)	0 (0.00%)	7 (100.00%)
Zululand	KZN263	6 (0.19%)	0 (0.00%)	6 (100.00%)
Zululand	KZN265	22 (0.70%)	0 (0.00%)	22 (100.00%)
Zululand	KZN266	14 (0.45%)	1 (7.14%)	13 (92.86%)
uMkhanyakude	KZN271	97 (3.10%)	4 (4.12%)	93 (95.88%)
uMkhanyakude	KZN272	70 (2.23%)	10 (14.29%)	60 (85.71%)
uMkhanyakude	KZN275	16 (0.51%)	1 (6.25%)	15 (93.75%)
uMkhanyakude	KZN276	7 (0.22%)	0 (0.00%)	7 (100.00%)
King Cetshwayo	KZN281	5 (0.16%)	0 (0.00%)	5 (100.00%)
King Cetshwayo	KZN282	239 (7.63%)	20 (8.37%)	219 (91.63%)
King Cetshwayo	KZN284	49 (1.56%)	4 (8.16%)	45 (91.84%)
King Cetshwayo	KZN285	19 (0.61%)	2 (10.53%)	17 (89.47%)
King Cetshwayo	KZN286	25 (0.80%)	3 (12.00%)	22 (88.00%)
iLembe	KZN292	122 (3.89%)	12 (9.84%)	110 (90.16%)
iLembe	KZN293	1 (0.03%)	0 (0.00%)	1 (100.00%)
Harry Gwala	KZN433	30 (0.96%)	5 (16.67%)	25 (83.33%)
Harry Gwala	KZN434	11 (0.35%)	1 (9.09%)	10 (90.91%)
Harry Gwala	KZN435	10 (0.32%)	1 (10.00%)	9 (90.00%)
Harry Gwala	KZN436	12 (0.38%)	3 (25.00%)	9 (75.00%)

GRT, genotypic resistance test; HIVDR, human immunodeficiency virus drug resistance; no., number
