# Supplementary material for: Patterns of HIV-1 Drug Resistance Observed Through Geospatial Analysis of Routine Diagnostic Testing in KwaZulu-Natal, South Africa
Source: Viruses. 2024 Oct 19;16(10):1634. doi: 10.3390/v16101634 (PMC11512327; doi:10.3390/v16101634)
Supplement: Supplementary file 1 [file viruses-16-01634-s001.zip › Supplementary Table S2.pdf]

Supplementary Table S2. Patterns of antiretroviral drug class resistance found in 3133 genotypic resistance tests from patients in KwaZulu-Natal province, South Africa.

Drug-class resistance patterns	All N=3133	2018 N=597	2019 N=675	2020 N=763	2021 N=666	2022[a] N=432
	**# GRTs; % (95% CI)**	**# GRTs; % (95% CI)**	**# GRTs; % (95% CI)**	**# GRTs; % (95% CI)**	**# GRTs; % (95% CI)**	**# GRTs; % (95% CI)**
**No resistance**						
PI/NRTI/NNRTI**	396; 12.64 (11.50-13.85)	75; 12.56 (10.01-15.49)	78; 11.56 (9.24-14.21)	115; 15.07 (12.61-17.81)	71; 10.66 (8.42-13.26)	57; 13.19 (10.15-16.75)
PI/NRTI/NNRTI/INSTI	2; 0.06 (0.01-0.23)	0; 0.00 (0.00-0.62)*	0; 0.00 (0.00-0.55)*	0; 0.00 (0.00-0.48)*	1; 0.15 (0.00-0.83)	1; 0.23 (0.01-1.28)
**Single-class resistance**						
INSTI	1; 0.03 (0.00-0.18)	0; 0.00 (0.00-0.62)*	0; 0.00 (0.00-0.55)*	0; 0.00 (0.00-0.48)*	0; 0.00 (0.00-0.55)*	1; 0.23 (0.01-1.28)
PI**	17; 0.54 (0.32-0.87)	7; 1.17 (0.47-2.40)	1; 0.15 (0.00-0.82)	3; 0.39 (0.08-1.14)	2; 0.30 (0.04-1.08)	4; 0.93 (0.25-2.35)
NRTI**	180; 5.75 (4.96-6.62)	32; 5.36 (3.69-7.48)	36; 5.33 (3.76-7.31)	45; 5.90 (4.33-7.81)	35; 5.26 (3.69-7.23)	32; 7.41 (5.12-10.30)
NNRTI	6; 0.19 (0.07-0.42)	1; 0.17 (0.00-0.93)	0; 0.00 (0.00-0.55)*	1; 0.13 (0.00-0.73)	2; 0.30 (0.04-1.08)	2; 0.46 (0.06-1.66)
NNRTI**	379; 12.10 (10.98-13.29)	79; 13.23 (10.62-16.22)	71; 10.52 (8.31-13.08)	101; 13.24 (10.91-15.85)	77; 11.56 (9.23-14.24)	51; 11.81 (8.92-15.23)
**Dual-class resistance**						
NRTI & INSTI	1; 0.03 (0.00-0.18)	0; 0.00 (0.00-0.62)*	0; 0.00 (0.00-0.55)*	0; 0.00 (0.00-0.48)*	0; 0.00 (0.00-0.55)*	1; 0.23 (0.01-1.28)
PI & NRTI	1; 0.03 (0.00-0.18)	0; 0.00 (0.00-0.62)*	0; 0.00 (0.00-0.55)*	0; 0.00 (0.00-0.48)*	1; 0.15 (0.00-0.83)	0; 0.00 (0.00-0.85)*
PI & NRTI**	126; 4.02 (3.36-4.77)	24; 4.02 (2.59-5.92)	29; 4.30 (2.90-6.11)	20; 2.62 (1.61-4.02)	34; 5.11 (3.56-7.06)	19; 4.40 (2.67-6.78)
PI & NNRTI**	8; 0.26 (0.11-0.50)	1; 0.17 (0.00-0.93)	2; 0.30 (0.04-1.07)	2; 0.26 (0.03-0.94)	3; 0.45 (0.09-1.31)	0; 0.00 (0.00-0.85)*
NRTI & NNRTI	7; 0.22 (0.09-0.46)	0; 0.00 (0.00-0.62)*	0; 0.00 (0.00-0.55)*	3; 0.39 (0.08-1.14)	2; 0.30 (0.04-1.08)	2; 0.46 (0.06-1.66)
NRTI & NNRTI**	1027; 32.78 (31.14-34.46)	188; 31.49 (27.78-35.39)	229; 33.93 (30.36-37.64)	242; 31.72 (28.43-35.15)	233; 34.98 (31.36-38.74)	135; 31.25 (26.90-35.85)
**Triple-class resistance**						
NRTI, NNRTI & INSTI	6; 0.19 (0.07-0.42)	0; 0.00 (0.00-0.62)*	0; 0.00 (0.00-0.55)*	1; 0.13 (0.00-0.73)	3; 0.45 (0.09-1.31)	2; 0.46 (0.06-1.66)
PI, NRTI & NNRTI	8; 0.26 (0.11-0.50)	1; 0.17 (0.00-0.93)	3; 0.44 (0.09-1.29)	0; 0.00 (0.00-0.48)*	4; 0.60 (0.16-1.53)	0; 0.00 (0.00-0.85)*
PI, NRTI & NNRTI**	959; 30.61 (29.00-32.26)	188; 31.49 (27.78-35.39)	223; 33.04 (29.50-36.73)	228; 29.88 (26.65-33.27)	195; 29.28 (25.85-32.90)	125; 28.94 (24.70-33.46)
**All-class resistance**						
PI, NRTI, NNRTI & INSTI	9; 0.29 (0.13-0.54)	1; 0.17 (0.00-0.93)	3; 0.44 (0.09-1.29)	2; 0.26 (0.03-0.94)	3; 0.45 (0.09-1.31)	0; 0.00 (0.00-0.85)*

#, number of; CI, confidence interval; GRT, genotypic resistance test; INSTI, integrase strand transfer inhibitor; NNRTI, non-nucleoside reverse transcriptase inhibitor; NRTI, nucleoside reverse transcriptase inhibitor; PI, protease inhibitor.

[a] Data only include period from 1 January 2022 to 30 June 2022.

* 1-sided, 97.5% confidence interval.

** GRTs did not include HIV-1 *integrase* testing.
