# Supplementary material for: Patterns of HIV-1 Drug Resistance Observed Through Geospatial Analysis of Routine Diagnostic Testing in KwaZulu-Natal, South Africa
Source: Viruses. 2024 Oct 19;16(10):1634. doi: 10.3390/v16101634 (PMC11512327; doi:10.3390/v16101634)
Supplement: Supplementary file 1 [file viruses-16-01634-s001.zip › Supplementary Table S3.pdf]

Supplementary Table S3. Characteristics of nine patients on dolutegravir-based treatment with dolutegravir resistance in KwaZulu-Natal, South Africa.

ID	Age	Sex	Log_{10} VL	Year[a]	Regimen (with XTC)[b]	GSS	Mutations by drug class			
							INSTI	PI	NRTI	NNRTI
1	37	Female	5.49	2018	TDF+DRV/r+DTG	1.5	*T66A*<br>*Q95K*<br>*T97A*<br>E138K*<br>Y143R<br>*S147G*	M46I<br>I50V<br>*F53L*<br>V82A	M41L<br>*D67G*<br>M184V<br>T215Y<br>*K219N*	Y181C<br>H221Y
2	40	Female	NA	2021	AZT+ETR+DRV/r+DTG	3.5	N155H<br>*G163R*<br>*D232N*	V32I<br>M46I<br>I54V<br>L76V<br>V82A<br>I84V	D67N<br>*K70Q*<br>*V75M*	V106M<br>*E138A***
3	32	Female	4.74	2021	TDF+DTG	1	*E157Q*<br>R263K	None	*K70Q*<br>M184V	*A98G*<br>K103N<br>P225H
4	39	Male	6.02	2021	TDF+DRV/r+DTG	2	*H51Y*	M46I<br>I50V<br>T74P<br>V82A	M41L<br>M184V<br>T215F<br>*K219R*	Y181C<br>F227L<br>*K238T*
5	33	Male	4.72	2021	ABC+DTG	0	G118R<br>E138T*	None	K65R<br>Y115F<br>M184V	K103N<br>V106M<br>*E138A***
6	35	Male	3.20	2021	TDF+DTG	1	R263K	None	K65R<br>*L74I*<br>M184V	L100I<br>K103N<br>V106M<br>*E138Q***
7	32	Male	5.24	2022	TDF+DTG	1	R263K	None	K70E<br>M184V	None
8	36	Female	5.28	2022	AZT+DTG	0	T66I<br>G118R<br>E138K*	None	D67N<br>K70R<br>M184V<br>*T215I*<br>K219Q	*A98G*<br>K103N<br>P225H<br>*K238T*
9	55	Female	4.18	2022	AZT+DTG	0.5	*L74M*<br>G118R<br>E138K*	None	D67N<br>K70R<br>M184V<br>K219E	K103N<br>P225H

ABC, abacavir; AZT, zidovudine; DRV/r, darunavir with boosted ritonavir; DTG, dolutegravir; ETR, etravirine; INSTI, integrase strand transfer inhibitor; NA, not available; NNRTI, non-nucleoside reverse transcriptase inhibitor; NRTI, nucleoside reverse transcriptase inhibitor; PI, protease inhibitor; TDF, tenofovir disoproxil fumarate; VL, viral load; XTC, lamivudine or emtricitabine.

Italics indicate accessory or other non-major mutations as classified by the 2022 edition of the IAS–USA drug resistance mutations list in conjunction with Stanford HIV Drug Resistance Database.

[a] Year of sample collection for HIV-1 genotypic resistance testing.

[b] All regimens included either lamivudine or emtricitabine.

* Classified as a minor mutation in the 2022 edition of the IAS–USA drug resistance mutations list, but as a major mutation in Stanford HIV Drug Resistance Database.

** Classified as a major mutation to rilpivirine only, in the 2022 edition IAS–USA drug resistance mutations list.
