# Supplementary material for: Patterns of HIV-1 Drug Resistance Observed Through Geospatial Analysis of Routine Diagnostic Testing in KwaZulu-Natal, South Africa
Source: Viruses. 2024 Oct 19;16(10):1634. doi: 10.3390/v16101634 (PMC11512327; doi:10.3390/v16101634)
Supplement: Supplementary file 1 [file viruses-16-01634-s001.zip › Supplementary Table S4.pdf]

Supplementary Table S4. HIV-1 drug resistance mutations detected in genotypic resistance tests from patients in KwaZulu-Natal, South Africa.

PI (N=2735)		NRTI (N=2735)		NNRTI (N=2735)		INSTI (N=39[a])	
Mutation	No. GRTs (%)	Mutation	No. GRTs (%)	Mutation	No. GRTs (%)	Mutation	No. GRTs (%)
_L10F_	552; (20.18)	A62V	62; (2.27)	_V90I_	36; (1.32)	T66A*	1; (2.56)
_L10I_	80; (2.93)	M41L	364; (13.31)	_A98G_	444; (16.23)	T66I	1; (2.56)
_L10R_	0; (0.00)	K65E	2; (0.07)	L100I	67; (2.45)	T66K*	0; (0.00)
_L10V_	74; (2.71)	K65N	3; (0.11)	K101E	247; (9.03)	_L74M_	1; (2.56)
_V11I_	9; (0.33)	K65R	170; (6.22)	K101H	53; (1.94)	_E92G_	0; (0.00)
_K20M_	14; (0.51)	_D67E_	3; (0.11)	K101P	51; (1.86)	E92Q	0; (0.00)
_K20R_	408; (14.92)	_D67G_	121; (4.42)	K103N	1302; (47.61)	_T97A_	3; (7.69)
_K20T_	170; (6.22)	D67N	670; (24.50)	K103S	116; (4.24)	G118R	3; (7.69)
_L23I_	106; (3.84)	_T69del_	15; (0.55)	V106A	3; (0.11)	F121Y	0; (0.00)
_L24I_	190; (6.95)	T69ins	3; (0.11)	_V106I_	66; (2.41)	E138A*	1; (2.56)
D30N	0; (0.00)	K70E	106; (3.88)	V106M	423; (15.47)	E138K*	3; (7.69)
V32I	63; (2.30)	K70R	614; (22.45)	_V106T_	0; (0.00)	E138T*	1; (2.56)
_L33F_	216; (7.90)	_K70Q_	28; (1.02)	V108I	175; (6.40)	G140A*	2; (5.13)
_M36I_	0; (0.00)	_L74I_	78; (2.85)	_E138A**_	247; (9.03)	G140C*	0; (0.00)
_M36L_	0; (0.00)	L74V	46; (1.68)	_E138G**_	49; (1.79)	G140R	0; (0.00)
_M36V_	0; (0.00)	_V75A_	1; (0.04)	_E138K**_	27; (0.99)	G140S*	0; (0.00)
_K43T_	77; (2.82)	V75I	58; (2.12)	_E138Q**_	52; (1.90)	Y143C	0; (0.00)
M46I	707; (25.85)	_V75M_	75; (2.74)	_E138R**_	2; (0.07)	Y143H	0; (0.00)
M46L	81; (2.96)	_V75T_	12; (0.44)	_V179D_	171; (6.25)	Y143R	2; (5.13)
I47A	51; (1.86)	_V75S_	0; (0.00)	_V179F_	5; (0.18)	_Y143S_	0; (0.00)
I47V	34; (1.24)	F77L	20; (0.73)	_V179L**_	3; (0.11)	S147G	1; (2.56)
_G48M_	0; (0.00)	Y115F	74; (2.71)	_V179T_	21; (0.77)	Q148H	0; (0.00)
G48V	7; (0.26)	F116Y	11; (0.40)	Y181C	218; (7.97)	Q148K	0; (0.00)
I50L	66; (2.41)	Q151M	15; (0.55)	Y181I	4; (0.15)	Q148R	2; (5.13)
I50V	41; (1.50)	M184V	2181; (79.74)	Y181V	7; (0.26)	_S153F_	0; (0.00)
_F53L_	50; (1.83)	M184I	13; (0.48)	Y188C	15; (0.55)	_S153Y_	0; (0.00)
_F53Y_	3; (0.11)	L210W	60; (2.19)	Y188H	6; (0.22)	N155H	5; (12.82)
_I54A_	12; (0.44)	_T215C_	2; (0.07)	Y188L	192; (7.02)	_S230R_	0; (0.00)
I54L	20; (0.73)	_T215D_	2; (0.07)	G190A	431; (15.76)	R263K	4; (10.26)
I54M	0; (0.00)	_T215E_	1; (0.04)	_G190E_	11; (0.40)		
_I54S_	0; (0.00)	T215F	238; (8.70)	G190S	24; (0.88)		
_I54T_	0; (0.00)	_T215I_	106; (3.88)	_H221Y**_	111; (4.06)		
I54V	719; (26.29)	_T215S_	6; (0.22)	P225H	517; (18.90)		
Q58E	163; (5.96)	_T215V_	17; (0.62)	F227C	0; (0.00)		
_I62V_	0; (0.00)	T215Y	140; (5.12)	_F227I_	1; (0.04)		
_H69K_	0; (0.00)	K219E	302; (11.04)	F227L	158; (5.78)		
_H69R_	0; (0.00)	_K219N_	14; (0.51)	_F227R_	0; (0.00)		
_A71T_	21; (0.77)	K219Q	340; (12.43)	_F227V_	0; (0.00)		
_A71V_	206; (7.53)	_K219R_	103; (3.77)	M230I	0; (0.00)		
_G73A_	1; (0.04)			M230L	36; (1.32)		
_G73C_	3; (0.11)			_L234I_	5; (0.18)		
_G73S_	34; (1.24)			_Y318F***_	0; (0.00)		
_G73T_	1; (0.04)						
T74P	57; (2.08)						
L76V	385; (14.08)						
_V77I_	0; (0.00)						
V82A	757; (27.68)						
_V82C_	36; (1.32)						
V82F	3; (0.11)						
V82L	13; (0.48)						
_V82M_	6; (0.22)						
V82S	6; (0.22)						
V82T	5; (0.18)						
N83D	10; (0.37)						
_I84A_	0; (0.00)						
_I84C_	0; (0.00)						
I84V	137; (5.01)						
_I85V_	4; (0.15)						
_N88D_	4; (0.15)						
N88S	29; (1.06)						
_L89I_	24; (0.88)						
_L89M_	27; (1.00)						
_L89V_	8; (0.29)						
L90M	83; (3.03)						

GRT, genotypic resistance test; INSTI, integrase strand transfer inhibitor; NNRTI, non-nucleoside reverse transcriptase inhibitor; No., Number; NRTI, nucleoside reverse transcriptase inhibitor; PI, protease inhibitor.

Italics indicate accessory or other non-major mutations as classified by the 2022 edition of the IAS–USA drug resistance mutations list in conjunction with Stanford HIV Drug Resistance Database.

[a] Only 39 out of 2735 GRTs with resistance had INSTI testing done in addition to PI, NRTI and NNRTI testing.

* Classified as a minor mutation in the 2022 edition of the IAS–USA drug resistance mutations list, but as a major mutation in Stanford HIV Drug Resistance Database.

** Classified as a major mutation to rilpivirine only, in the 2022 edition IAS–USA drug resistance mutations list.

*** Classified as a major mutation to doravirine only, in the 2022 edition IAS–USA drug resistance mutations list.
